# Genomic dynamics of high-risk carbapenem-resistant *Klebsiella pneumoniae* clones carrying hypervirulence determinants in Egyptian clinical settings

**DOI:** 10.1186/s12879-024-10056-1

**Published:** 2024-10-22

**Authors:** Nehal Adel Abdelsalam, Shahira A. ElBanna, Shaimaa F. Mouftah, José F. Cobo-Díaz, Ahmed H. Shata, Sherine M. Shawky, Reham Atteya, Mohamed Elhadidy

**Affiliations:** 1 Center for Genomics, Helmy Institute for Medical Sciences, Zewail City of Science and Technology, Giza, Egypt; 2 Biomedical Sciences Program, University of Science and Technology, Zewail City of Science and Technology, Giza, Egypt; 3 Department of Microbiology and Immunology, Faculty of Pharmacy, Cairo University, Cairo, Egypt; 4 Department of Food Hygiene and Technology, Institute of Food Science and Technology, Universidad de León, León, Spain; 5 Department of Microbiology, Medical Research Institute, Alexandria University, Alexandria, Egypt; 6 Department of Bacteriology, Mycology and Immunology, Faculty of Veterinary Medicine, Mansoura University, Mansoura, Egypt

**Keywords:** *Klebsiella pneumoniae*, Convergence, ESBL, Carbapenem, Hypervirulence genetic determinants, Extensive-drug resistance

## Abstract

**Background:**

Ongoing studies have revealed the global prevalence of severe infections caused by the hypervirulent strains of *Klebsiella pneumoniae (K. pneumoniae)*. Meanwhile, the World Health Organization and the Centers for Disease Control declared carbapenem-resistant *K. pneumoniae* as an urgent public health threat, requiring swift and effective action to mitigate its spread. Low- and middle-income countries are severely impacted by such devastating infectious diseases owing to the ill implementation of antimicrobial practices and infection control policies. Having both hypervirulence and carbapenemase gene determinants, the emergence of convergent hypervirulent carbapenem-resistant *K. pneumoniae* is now being reported worldwide.

**Methods:**

In this study, we sequenced 19 carbapenemase-producing *K. pneumoniae* strains recovered from various clinical specimens. Additionally, we evaluated the phenotypic antimicrobial susceptibility to multiple antimicrobial classes using the VITEK2 automated system. Utilizing the sequencing data, we characterized the sequence types, serotypes, pangenome, resistance profiles, virulence profiles, and mobile genetic elements of the examined isolates. We highlighted the emergence of high-risk clones carrying hypervirulence genetic determinants among the screened isolates.

**Results:**

Our findings revealed that all carbapenem-resistant isolates exhibited either extensive- or pan-drug resistance and harbored multiple variants of resistance genes spanning nearly all the antimicrobial classes. The most prevalent carbapenemase genes detected within the isolates were *bla*_*NDM−5*_ and *bla*_*OXA−48*_. We identified high-risk clones, such as ST383-K30, ST147-K64, ST11-K15, and ST14-K2, which may have evolved into putative convergent strains by acquiring the full set of hypervirulence-associated genetic determinants (*iucABCD*, *rmpA* and/ or *rmpA2*, putative transporter *peg-344*). Additionally, this study identified ST709-K9 as a high-risk clone for the first time and uncovered that capsule types K15 and K9 carried hypervirulence genetic determinants. The most frequent Inc types found in these isolates were Col440I, IncHI1B, and Inc FII(K).

**Conclusion:**

This study highlights the emergence of high-risk, extensively carbapenem-resistant *K. pneumoniae* strains co-carrying hypervirulence determinants in Egyptian clinical settings. This poses an imminent threat not only to Egypt but also to the global community, underscoring the urgent need for enhanced surveillance and control strategies to combat this pathogen.

**Supplementary Information:**

The online version contains supplementary material available at 10.1186/s12879-024-10056-1.

## Introduction

*Klebsiella pneumoniae (K. pneumoniae*) is a prominent member of the ESKAPE pathogens, a group of bacteria recognized for their ability to develop antimicrobial resistance (AMR) [1]. *K. pneumoniae* is a human commensal and an opportunistic pathogen that exhibit dual potential for causing community- and hospital-acquired infections [2]. *K. pneumoniae* can progress from colonization to infection due to host-associated risk factors, including microbiome dysbiosis, immunocompromise, endoscopy procedure, alcoholism, cancer, and diabetes [3, 4].

The dynamic genome of *K. pneumoniae* facilitates its rapid acquisition of resistance to multiple classes of antimicrobials, such as beta-lactams, fluoroquinolones, and aminoglycosides, particularly within clinical contexts [5]. Thus, multi-drug resistant (MDR) *K. pneumoniae* has become an inevitable global threat to the public health [6, 7]. The emergence of carbapenem-resistant *K. pneumoniae* further complicates treatment strategies, as carbapenems are often considered the last safe antimicrobial against these infections.

Besides its growing AMR profile, the pathogenicity of *K. pneumoniae* is attributed to a diverse arsenal of virulence factors that contribute to the severity of infection, including polysaccharide capsule, lipopolysaccharides, fimbriae, and siderophores [8]. The acquisition of particular virulence determinants, *rmpA/rmpA2* hypermucoviscosity genes, specific polysaccharide capsule type, aerobactin siderophore, and putative transporter *peg-344*, renders some *K. pneumoniae* strains to be described as hypervirulent [9]. Notably, infections caused by hypervirulent *K. pneumoniae* are rising globally and contribute to higher mortality rate [9]. While hypervirulent strains of *K. pneumoniae* are genetically distinct from classical strains, there are recent reports of infections caused by MDR hypervirulent *K. pneumoniae* [10]. This phenomenon is known as convergent adaptation or convergence that occurs when either a MDR *K. pneumoniae* strain acquires hypervirulence genes, a hypervirulent *K. pneumoniae* strain acquires AMR genes, or a strain acquires a mosaic plasmid carrying both hypervirulence and resistance genes [11].

Detailed genomic data on the prevalence of convergent hypervirulent *K. pneumoniae* strains remain limited. The objective of this study is to implement high-throughput sequencing and genomic analysis to identify epidemiological and molecular biomarkers of adaptation in MDR *K. pneumoniae* carrying hypervirulence genetic determinants across different clones in Egyptian clinical settings. Thus, the study advances genomic surveillance of severe infections caused by putative convergent hypervirulent, and carbapenem-resistant *K. pneumoniae*.

## Methods

### Bacterial isolates collection

Forty-six clinical non-duplicate isolates of *K. pneumoniae* were collected from a clinical microbiology laboratory serving multiple hospitals in Alexandria, Egypt between August 2020, and April 2021 [12]. To ensure diversity and avoid related isolates, we used random sampling approach based on the month of isolation and source. The isolates were recovered from various sources, including ascitic fluid, wound, blood, bronchoalveolar lavage, sputum, cerebrospinal fluid, and urine. Patient identities were kept confidential, and no patient data were collected. The isolates were historical and collected before the study period, so both ethics approval and patient consent were waived.

The isolates were selected based on their clinical relevance, specifically those linked to infections and exhibiting multidrug resistance, with a particular focus on carbapenem-resistant strains. These isolates were collected during routine diagnostic procedures and were all confirmed as carbapenem-resistant using the VITEK 2 system (bioMérieux, Marcy L’Étoile, France). For sequencing purposes, inclusion criteria for clinical *K. pneumoniae* isolates to be sequenced were carbapenemase production and phenotypic resistance to meropenem and imipenem. Twenty-eight isolates fulfilling these criteria were randomly chosen for short-read sequencing.

### Antimicrobial susceptibility testing

The isolates were cultured on Mueller–Hinton agar overnight at 37 °C for phenotypic antimicrobial susceptibility testing using VITEK 2 (bioMérieux, Marcy L’Étoile, France) AST-N222 card. The antimicrobial classes included penicillin (ticarcillin, piperacillin, ticarcillin/clavulanic acid, piperacillin/tazobactam), as well as cephalosporins (ceftazidime, cefepime), monobactams (aztreonam), carbapenems (imipenem, meropenem), aminoglycosides (amikacin, gentamicin, tobramycin), quinolones (ciprofloxacin), and folate pathway antagonists (trimethoprim/sulfamethoxazole). The minimum inhibitory concentrations (MIC) breakpoints were interpreted according to the global Clinical and Laboratory Standards Institute guidelines [13].

### *K. pneumoniae* whole genome sequencing and assembly

Whole genome sequencing of the isolates was carried out as published in our previous study [14]. Briefly, 1 µg of genomic DNA and the NEXTflex Rapid XP DNA-Seq kit was used for library preparation (PerkinElmer, https://perkinelmer-appliedgenomics.com/). NextSeq 500/550 mid output kit v2.5 was used for sequencing. Raw reads resulted were paired-end and short-reads that were submitted to NCBI Bioproject database under the accession number PRJNA906139. Biosamples and Sequence Read Archive accessions were provided in the supplementary table S1, additional file 1. Raw reads were filtered to remove adaptors and low-quality reads with phred score less than 15 using fastp default settings [15]. Subsequently, the filtered reads underwent de novo assembly into contigs using Unicycler (v0.4.8) [16]. The quality of the assembled contigs was assessed by QUAST [17]. CheckM [18] was used to assess the completeness and contamination of the assembled contigs. Only 19 isolates genomes were 90% or more complete and less than 5% contaminated and thus were included in the study for further analysis.

### Taxonomy, multi-locus sequence typing, serotyping, and annotation

To confirm the taxonomy of the isolates, KmerFinder (https://www.genomicepidemiology.org/) were employed for the analysis of the filtered reads [19]. For the determination of multi-locus sequence types (MLST) of the isolates, contigs were analyzed using BIGSdb-Pasteur (https://bigsdb.pasteur.fr/) and MLST 2.0 software at the Center for Genomic Epidemiology (https://www.genomicepidemiology.org/). All the isolates contigs were typed for capsule polysaccharide (CPS, K-typing) and lipopolysaccharide (LPS, O-typing) using Kaptive [20].

Isolates contigs were annotated using Prokka [21]. The GFF3 output files from Prokka were input into Roary for the quantification of core, shell, and cloud genes [22].

### Characterization of resistance determinants

Isolates contigs were analyzed by Abricate (https://github.com/tseemann/abricate) using CARD as the antimicrobial resistance (AMR) database and filtered reads were analyzed using ResFinder [23] (http://genepi.food.dtu.dk/), for AMR genes. PointFinder [24] was used to detect chromosomal point mutations. Resistance genes retrieved by CARD and ResFinder databases had a minimum of 80% identity and 80% coverage. Additionally, the isolates contigs were scanned using the Institut Pasteur database (https://bigsdb.pasteur.fr/klebsiella/) to detect heavy metal resistance genes.

### In-Silico analysis of virulence and hypervirulence genetic determinants

To identify virulence genes among isolates, their genomes were screened using Abricate against Virulence Factor database (VFDB) with a minimum of 60% coverage and 80% identity. Virulence genes were categorized based on the VFDB classification, with special emphasis on hypervirulence gene determinants. These determinants included the full siderophore aerobactin gene cluster *iucABCD*, the hypercapsulation genes *rmpA/A2*, and putative transporter *peg-344*.

The putative transporter *peg-344* was detected by searching the isolates contigs using the nucleotide Basic Local Alignment Search Tool (BLAST) [25]. The sequence of the putative transporter *peg-344* was retrieved from GenBank database (accession MW911667.1). An isolate was reported as putative hypervirulent when it carried the full gene set of *iucABCD*,* rmpA* and/ or *rmpA2*, and the putative transporter *peg-344.*

### Characterization of mobile genetic elements

Isolates contigs were screened for plasmids using Platon software [26]. The extracted plasmid contig sequences were then validated using mlplasmids software [27]. Only validated plasmid contig sequences were considered for further analysis. These validated sequences were screened for AMR and virulence genes using Abricate with ResFinder and VFDB, respectively. Plasmid annotation was performed with MobileElementFinder (v1.0.3) (https://cge.food.dtu.dk/services/MobileElementFinder/) using thresholds of 80% coverage and 90% identity. Mobilizable plasmids were identified using MOB-typer [28].

To detect the insertion sequences and composite transposons, the contigs of the isolates were used as input for MobileElementFinder, which is based on ISFinder database [29]. Integrons were detected by Integron Finder 2.0 software to report promotor, *attI*, and provide functional annotation [30].

To visualize the mobile genetic elements, Proksee [31] webtool was used. Mobile genetic elements were annotated and visualized when it had 80% identity and 80% coverage against mobileOG-db [32].

### Phylogenomic tree construction and visualization

To integrate MDR and hypervirulent strains of *K. pneumoniae* in the phylogenomic analysis, the complete genomes of *K. pneumoniae subsp. pneumoniae* HS11286 (GCF_000240185.1) and *K. pneumoniae subsp. pneumoniae* NTUH-K2044 (GCF_000009885.1) were retrieved from NCBI Genome database (https://www.ncbi.nlm.nih.gov/datasets/genome/), respectively. Core genome multi-locus sequence typing (cgMLST) was then conducted using fast-GeP, where NTUH-K2044 genome was set as a reference [33]. The core genome polymorphic genes (genes with at least one nucleotide difference among all genomes) were selected and concatenated by the ruby script concat_cgMLST_genes.rb (https://github.com/JoseCoboDiaz/concat_cgMLST_genes). The concatenated gene-by-gene fasta file was used for alignment and phylogenomic tree building using MAFFT version 7 [34]. The parameters were set as default parameters for alignment, the Neighbor-Joining method, the Jukes-Cantor substitution model, and 1000 bootstrap resampling. The cgMLST tree was visualized using iTOL (https://itol.embl.de/) [35].

## Results

### Phenotypic susceptibility to antimicrobials

All the 19 isolates included in the study were originally classified as carbapenemase-producing based on the modified carbapenem inactivation method [12] and carbapenem-resistant based on VITEK 2 results. All the isolates were resistant to beta-lactams, including penicillins and cephalosporins, as well as carbapenem antibiotics. Only 21% of isolates (4/19) were susceptible to aztreonam (MIC < = 1). Concerning the aminoglycosides class, all the isolates were resistant to tobramycin (MIC > = 8), while only 10% (2/19) were susceptible to both amikacin (MIC < = 4) and gentamicin (MIC < = 1). Additionally, 10% of the isolates (2/19) showed intermediate sensitivity to gentamicin (MIC = 4). Resistance to ciprofloxacin (MIC > = 2) was observed in all the isolates, while approximately 79% (15/19) showed resistance to trimethoprim/sulfamethoxazole combination (MIC > = 80).

Based on the above results, approximately 58% of the isolates (11/19) were identified to be pan-drug resistant (PDR), while 42% (8/19) were classified as extensively-drug resistant (XDR) as previously defined [36]. The MIC values for each antimicrobial and their corresponding interpretation were provided in supplementary table S2, additional file 1.

### Assembly statistics

The average assembly size of the isolates was 5.6 Mbp, with a GC content of 56–57%. The number of contigs ranged from 94 to 1170. While the N50 varied between 5991 and 131,359, the L50 values ranging from 15 to 239. Only 19 isolates’ genomes were at least 90% complete and had contamination levels of less than 5% and were included in the study.

### Typing and pangenome analysis of *K. pneumoniae*

In this study, the most prevalent sequence types (STs) were ST383 and ST147, followed by ST11. The STs of two isolates could not be identified due to the incomplete coverage of one or more alleles. No sequence type showed a specific association with isolation source, however, ST383 was predominantly isolated from the respiratory tract, with fewer occurrences in wound samples. A significant association between capsule polysaccharide K-type and sequence type was observed among the isolates. The most frequent K-type identified were K30 and K64, followed by K15 and K9 serotypes. Lipopolysaccharide O-type was less diverse compared to K-type, where O1 was predominant.

The pangenome analysis revealed 2,377 core genes present in all isolates and 4,374 shell genes present in 15-95% of the isolates. Additionally, 3,192 cloud genes were found in less than 15% of the isolates.

### Distribution of antimicrobial resistance genes across isolates

**Fig. 1:**
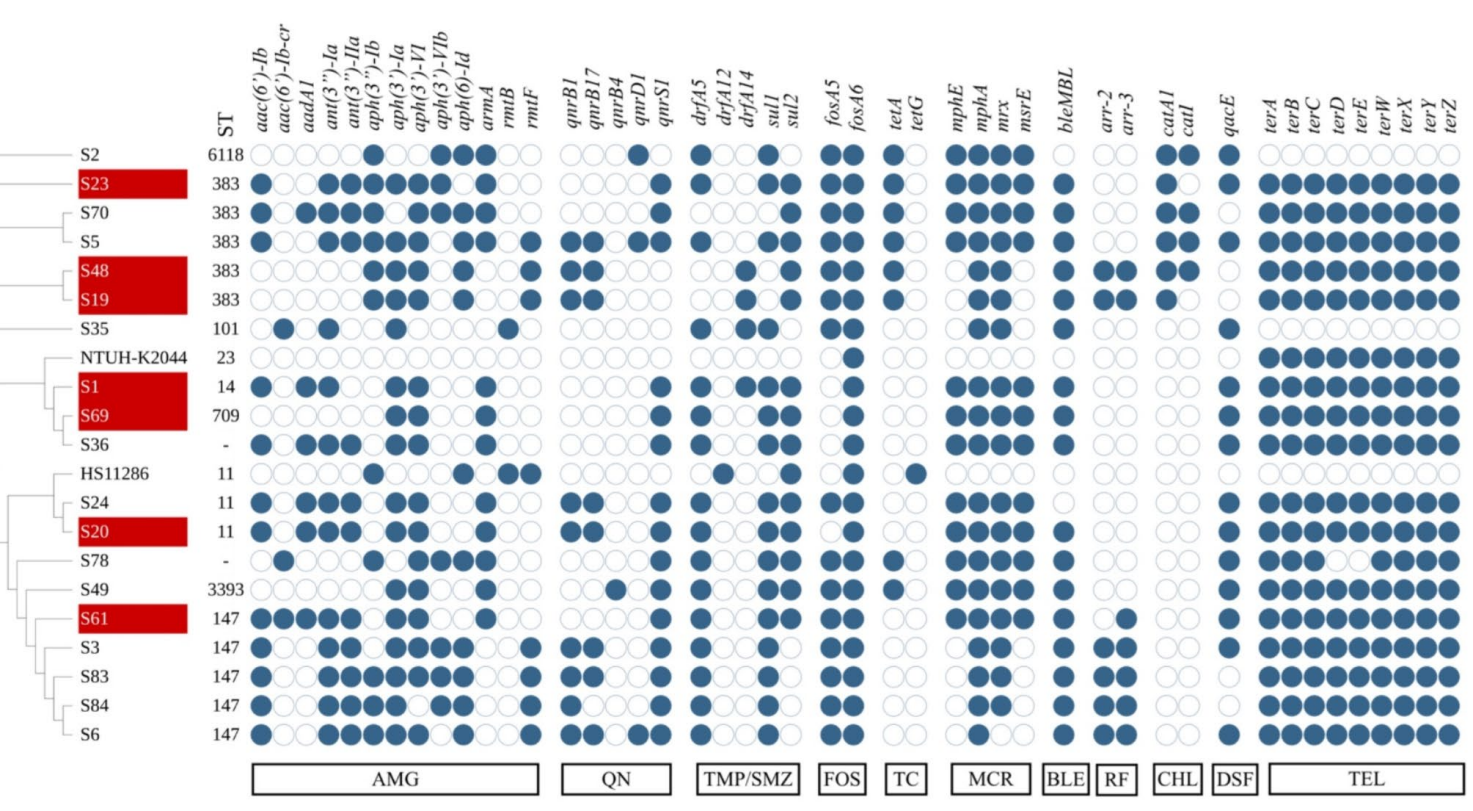
cgMLST phylogenomic analysis of the 19 isolates and the distribution of sequence types and resistance genes. S denotes sample. Potential convergent isolates are highlighted in dark red. Genes included aminoglycosides (AMG), quinolones (QN), trimethoprim/sulfamethoxazole (TMP-SMZ), fosfomycin (FOS), tetracycline (TC), macrolides (MCR), bleomycin (BLE), rifampin (RF), chloramphenicol (CHL), disinfectants (DSF), and tellurite (TEL). Colored circles indicate gene presence. Colorless circles indicate gene absence. The figure was created using iTOL

All isolates showed a high prevalence of AMR genes, conferring resistance to most of the available antimicrobial classes. These classes included β-lactams, carbapenems, aminoglycosides, quinolones, trimethoprim/sulfamethoxazole combination, fosfomycin, tigecycline, macrolide, chloramphenicol, and rifampicin (Fig. 1).

### Extended-spectrum β-lactamase (ESBL) and carbapenemase genes and their co-occurrence

**Fig. 2:**
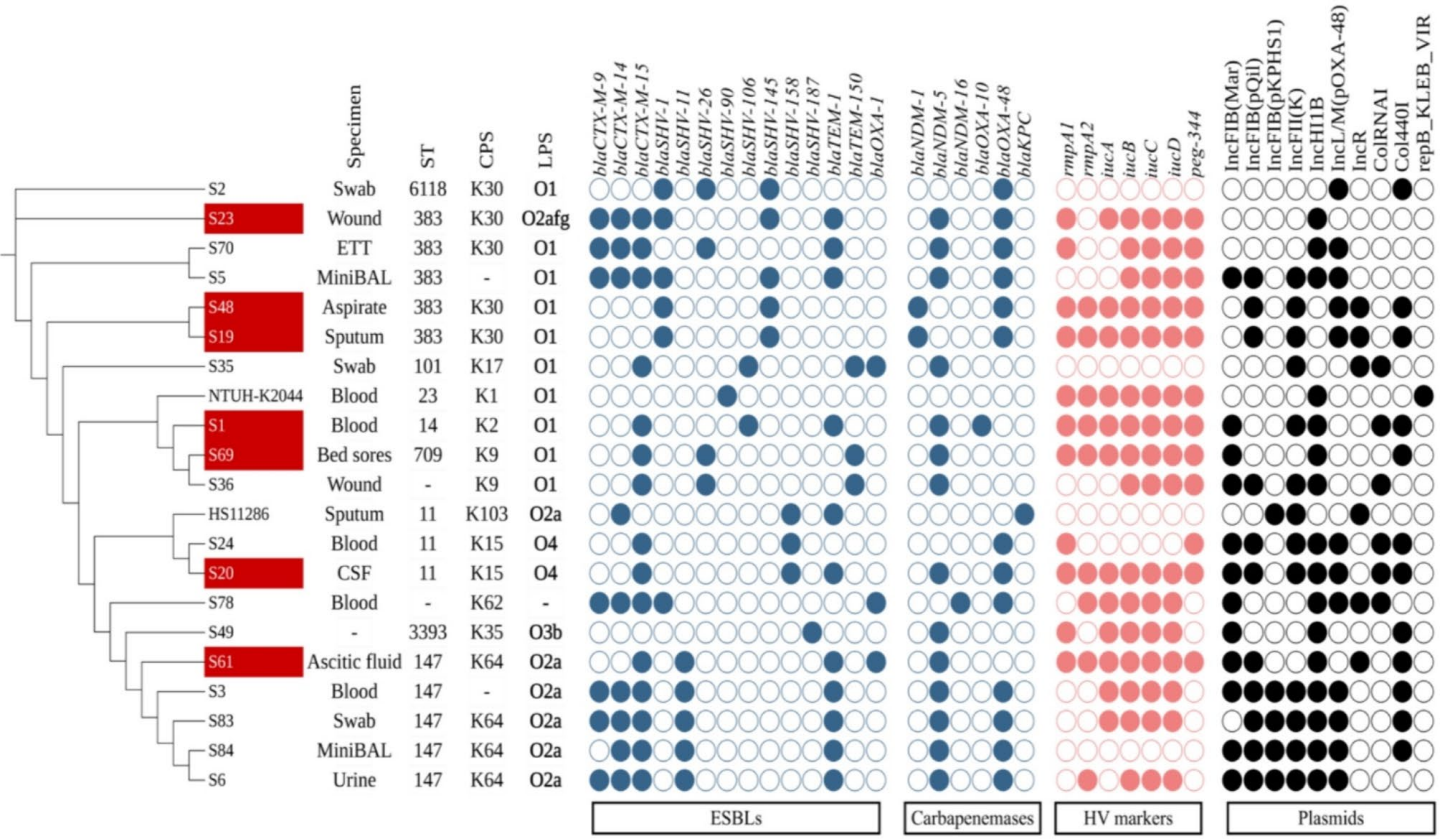
cgMLST phylogenomic analysis with distribution of STs, capsule type, lipopolysaccharide type, ESBLs and carbapenemases genes. S denotes sample. Potential convergent isolates are highlighted in dark red. ETT: Endotracheal tube; BAL: Bronchoalveolar lavage; CSF: Cerebrospinal fluid; CPS: Capsule polysaccharide; LPS: Lipopolysaccharide; ESBL: Extended spectrum beta-lactamase; HV: Putative hypervirulence. Colored circles indicate gene presence. Colorless circles indicate gene absence. The figure was created using iTOL

Nearly all the isolates exhibited resistance to extended-spectrum β-lactam antibiotics, aztreonam, and penicillins, including their combinations. Genotypic analysis, consistent with phenotypic results, revealed multiple ESBL genes in the isolates (Fig. 2). The *bla*_CTX−M−15_ was the most predominant ESBL gene followed by *bla*_TEM−1_. Notably, *bla*_SHV_ exhibited considerable variability and certain variants were more prevalent than others. Multiple isolates (15/19) harbored combinations of *bla*_CTX_, *bla*_TEM_, and *bla*_SHV_. Notably, the narrow spectrum oxacillinase *bla*_OXA−9_ was detected in 63% of the isolates (12/19).

Among the carbapenemase genes, *bla*_NDM−5_ and *bla*_OXA−48_ were the most frequent variants found in the isolTES. 42% of the isolates (8/19) carried both *bla*_NDM−5_ and *bla*_OXA−48_ carbapenemase genes. None of the isolates carried either *bla*_KPC_, *bla*_GES_, *bla*_IMP,_*bla*_VIM,,_ or *bla*_GIM_ carbapenemase genes. Notably, all isolates were found to carry a combination of one or more ESBL and carbapenemase genes.

Upon further investigation of known chromosomal mutation contributing to a decreased carbapenem susceptibility, almost all the isolates (18/19) were found to harbor two different point mutations in the outer membrane protein *OmpK37* gene (I70M and I128M), particularly associated with resistance to beta-lactam antibiotics. Two isolates belonging to ST11 harbored an additional point mutation N230G. Other mutations in OmpK35 included a premature stop codon at positions 89 (E89*) and 316 (W316*), and a frameshift at position 57 (T57delinsTYA). In the *OmpK36* genes, multiple premature stop codons were detected at positions 140, 236, and 243. Fewer premature stop codons and frameshift mutations were found in the *OmpK37* gene.

### Aminoglycoside, quinolones, trimethoprim/sulfamethoxazole, and other resistance genes

All the isolates carried at least one resistance gene of aminoglycoside modifying enzymes class (Fig. 1). The *armA* was detected in multiple isolates followed by *rmtF* and *rmtB* as members of 16 S ribosomal RNA methyltransferases family.

*K. pneumoniae* isolates exhibited diverse resistance mechanisms to quinolones, including efflux pumps and plasmid-encoded resistance via *qnr* genes. Chromosomal mutations were found in *parC*, *gyrA*, and *acrR* genes. The most frequent mutation in the *gyrA* was S83F (37% ;7/19), followed by D87N mutation (32% ;6/19), and D87A *gyrA* mutation (11%;2/19). On the other hand, E84K mutation in *parC* was detected in 42% (8/19), and S80I mutation in 21% (4/19) of isolates. Almost all the isolates (95%; 18/19) carried various *acrR* mutations, including P161R, G164A, F172S, R173G, L195V, F197I, and K201M.

A total of 84% of the isolates (16/19) carried both *dfrA5* and *sul1*, conferring resistance to trimethoprim and sulfonamides, respectively. All isolates carried variants of the *fosA* gene, which encodes the fosfomycin-modifying enzyme. For tetracycline and tigecycline resistance, the tetracycline efflux pump gene, *tetA*, was most frequently observed among isolates. Regarding macrolides, all the isolates carried the macrolide 2’-phosphotransferase I *mphA* resistance gene. Macrolide inactivation gene cluster *mrx* gene, macrolide phosphotransferase *mphE*, and ABC-F subfamily *msrE* genes were also found. As a resistance mechanism against rifampin, both ribosyltransferase *arr-2* and *arr-3* genes were found in six isolates.

All screened isolates possessed *oqxA* and *oqxB*, encoding the *OqxAB* multi-drug efflux pump, which confers resistance to disinfectants, as well as diaminopyrimidine fluoroquinolone, glycylcycline, nitrofuran, and tetracycline.

On the heavy metals resistance level, one isolate carried mercury resistance gene cassette *merC*,* merP*,* merR*, and *merT* genes, arsenic resistance genes cassette (*arsABCDR*), and copper resistance genes cassette (*pcoABCDERS*), and silver resistance gene cassette (*silABCDFGPRS*). However, this isolate did not have any tellurite resistance genes. (Supplementary table S3, additional file 1).

### Genomic characterization virulence determinants

A myriad of virulence genes and gene clusters were identified among most of the isolates (supplementary table S4, additional file 1). All the isolates carried multiple regulatory genes, including CRP, *fur*, *phoP*, *rpoS*, and *rcsB*. Polysaccharide capsule formation is encoded by multiple genes detected in all screened isolates, such as *gndA*, *galF*, *orf2*, *wzi*, *manC*, and *wza*. Interestingly, more than 63% (12/19) of the isolates carried hypervirulence hypercapsulation encoding genes *rmpA* or *rmpA2* or both. The *rfbABDK1* gene cluster for lipopolysaccharide formation was fully present in approximately 63% of the isolates (12/19). Adherence is a significant virulence factor mediated by *Escherichia coli* common pilus (ECP) and type 1 fimbriae gene clusters among the studied isolates. All the screened isolates carried different ECP variants except for one isolate lacking *yagX*/*ecpC*. Likewise, the *fim* gene cluster, responsible for type 1 fimbriae structure, was present in all isolates. Type 3 fimbriae genes involved in biofilm formation were remarkably present in almost all the isolates and almost 74% of the isolates (14/19) possessed the full *mrk* gene cluster.

Among the siderophores and iron acquisition systems, approximately 21% of the isolates (4/19) harbored the *Klebsiella* ferric uptake *kfuA* and *kfuB.* Nearly all the isolates possessed the four known siderophore systems: enterobactin, aerobactin, salmochelin, and yersiniabactin gene clusters. Enterobactin was the most prevalent siderophore in the screened isolates, where the complete gene clusters for enterobactin biosynthesis (*entABCDEFS*) and transport (*fepABCDG*) were found. All isolates carried the enterobactin *fes* gene required for iron release. Regarding aerobactin, approximately 58% of the isolates (11/19) carried the hypervirulent marker *iucA*, in addition to the *iucBCD* gene cluster encoding aerobactin, and *iutA* for its transport. Approximately 89% of the isolates carried *iroE* gene (17/19), which encodes enzymes involved in the biosynthesis of salmochelin. The Yersinia high-pathogenicity island, responsible for synthesizing yersiniabactin siderophore, was present in more than 73% of isolates (14/19).

### Genomic characterization of mobile genetic elements

Among the known Inc types, isolates exhibited an average of five plasmids per isolate, with a maximum of eight and a minimum of two plasmids in each isolate genome. Among the most common plasmids detected were IncHI1B, IncL/M(pOXA-48), Col440I, IncFII(K), IncFIB(Mar), and IncFIB(pQil). In a descending order of frequency, relaxase types included MOBP, MOBH, MOBF, MOBC, and MOBQ. The MPF systems included types F, T, and I, where type F is notably the more prevalent (supplementary table S5, additional file 1).

Furthermore, our screened isolates harbored multiple insertion sequences (IS), ranging from seven IS and 16 IS per isolate (Supplementary table S6). The most prevalent IS families were IS3, IS1, IS6, and IS5. Multiple composite transposons (cn) were found common and unique within isolates genome. Specifically, cn_3079_ISEc33_IS630 were common in five isolates.

Class 1 integron was detected in 84% (16/19) of the isolates. Multiple drug and disinfectant resistance genes were carried on integrons, such as *qacE* (9/19), aminoglycosides resistance often coupled with *ant(3’’)-Ia* (7/19), *aac(6’)-Ib* (8/19), rifampin resistance *arr* (4/19), trimethoprim resistance *dfrA1* (5/19), ESBL *bla*_OXA−1_ (5/19), chloramphenicol resistance *catB* (2/19), and sulfonamide resistance *sul1* (1/19).

### Emergence of carbapenem-resistant *K. pneumoniae* isolates carrying hypervirulence genetic determinants

All the 19 isolates were either XDR or PDR carrying a plethora of AMR genes that included ESBLs and carbapenemase encoding genes. The resistant isolates that acquired the full gene set of hypervirulence determinants (*iucABCD*, *rmpA/A2*,* peg-344)* were reported in this study as potential convergent isolates. Both aerobactin *iucABCD* gene cluster and *rmpA/rmpA2* genes were detected on the same plasmid indicating their co-transfer. Consequently, there were a total of 7 potential convergent strains (highlighted in dark red in Fig. 2), each belonged to different sequence types and capsule types. ST383-K30 was the most frequent sequence type represented by 3 isolates, followed by single isolates representing each of the following strains: ST14-K2, ST11-K15, ST147-K64, and ST709-K9. Four of these isolates were serotyped as O1, while the rest of the isolates were either O2a, O2afg, or O4.

Given the observed high genome plasticity of these isolates in acquiring both AMR and virulence genes, their mobile genetic elements were investigated. Among these strains, Col440I was the most prevalent Inc types, followed by IncHI1B, IncFIB(Mar), IncFIB(pQil), and IncFII(K). Meanwhile, IncL/M(pMU407) was the least frequent Inc type, detected in only one strain (ST383-K30). While present in other isolates, two insertion sequences (IS), namely ISKpn21 and ISVsa3, were found in all the potential convergent isolates. These were followed by ISKpn19, IS102, and IS6100.

**Fig. 3:**
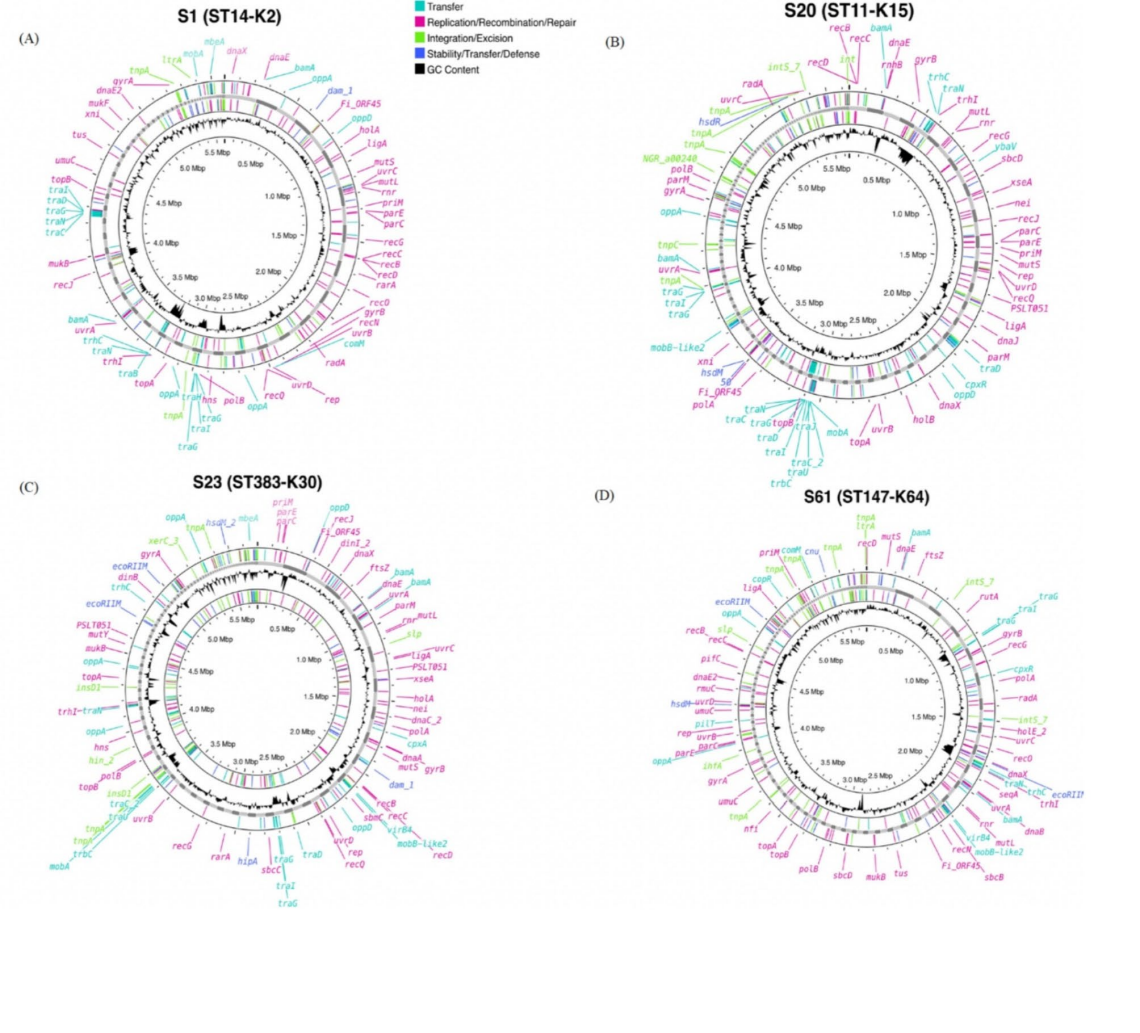
**A-D**: Determinants of MGE in high-risk isolates. Each determinant is colored according to MGE group

To further explore the mechanisms underlying the integration of mobile genetic elements (MGEs) into the potential convergent strains, the contigs of these strains were annotated by mobileOG-db (Fig. 3A and D, S1-S3). To excise and integrate a mobile genetic element into their genomes, the strains possessed *tnpA*,* tnpC*,* intS*,* slp*, and *ihfA* genes. Replication, recombination, and repair of mobile genetic elements held the highest number of genes compared to other categories. These included *dnaE*,* dnaC*,* dnaX*,* recBCDGJOQ* cluster, *gyrA*,* gyrB*,* parC*,* parE*,* uvrABCD* cluster, *holA*,* holB*,* mutL*,* mutS*, and others. For transfer, the most abundant genes were *mobA*,* mobB*,* traCDGINU* cluster, *trhC*,* trhW*,* oppD*,* oppF*,* cpxA*,* cpxR*,* trbC*,* tonB*,* bamA*,* trbC*,* mbeA*,* ygaD*, and *copR*. To stabilize MGEs, strains possessed *ecoRIIM*,* dam*,* hsdR*,* hsdM*, and *hipA.*

## Discussion

A significant increase in MDR and XDR *K. pneumoniae* has been documented following the COVID-19 pandemic [37]. The escalating AMR observed in *K. pneumoniae* can be attributed to the widespread misuse and self-medication of antibiotics during the pandemic in Egypt [12, 37]. Notably, up until 2019, no pan-drug resistant *K. pneumoniae* strains had been reported in the country [38]. Our data indicates that all isolates were either XDR or PDR to most antimicrobials, with only a small number showing sensitivity to aztreonam, amikacin, gentamicin, and the trimethoprim/sulfamethoxazole combination. This alarming trend signals a potential emergence of an untreatable endemic, posing a significant threat to the country.

Carbapenems, such as meropenem and imipenem, are effective against ESBL-producing *K. pneumoniae* and are considered almost the last safe resort for treating these infections. However, the widespread use of carbapenems lead to resistance, primarily due to the production of carbapenemase enzymes that most commonly include KPC, OXA, and NDM. The treatment of carbapenem-resistant *K. pneumoniae* infections poses significant challenges, mainly due to the nephrotoxic and neurotoxic side effects of the last effective antibiotic class, polymyxins. Carbapenem resistance is increasingly prevalent across various healthcare settings in Egypt [39].

The first reported instance of *bla*_NDM−1_, a carbapenemase gene, occurred in Egypt in a *K. pneumoniae* strain isolated from the gastric fluid sample of a cancer patient who had no history of travel outside Egypt [40]. Later, *bla*_NDM_ carbapenemase-producing *K. pneumoniae* were isolated from urine and sputum specimens collected from hospitalized patients [41]. *K. pneumoniae* has emerged as the most frequent carbapenem-resistant Gram-negative organism responsible for life-threatening late-onset sepsis in neonates, with *bla*_NDM−1_ and *bla*_OXA−48_ being the most common carbapenemase genes detected strains isolated from patients [42, 43]. Although *bla*_KPC_ carbapenemase was reported in multiple studies on *K. pneumoniae* [44–49] and found in MDR reference strain, it was not detected in any of the screened isolates. All the isolates exhibited phenotypic resistance to carbapenem carried either a variant of *bla*_NDM_ (*bla*_NDM−1_ or *bla*_NDM−5_) or *bla*_OXA_ (*bla*_OXA−10_ or *bla*_OXA−48_), or both. *bla*_NDM−1_ variant disseminated successfully according to the early epidemiological studies in Egypt [45, 50–52], and it was detected in only two putative convergent isolates in our current study. Conversely, *bla*_NDM−5_, a variant reported more recently and with lower frequency in Egypt [53, 54], was found to be more prevalent among our isolates and posed a greater resistance threat due to its more active hydrolysis of carbapenems compared to *bla*_NDM−1_ [55].

Meanwhile, *bla*_OXA−48_ continued to disseminate successfully in our study, regardless of isolate sequence type, and *bla*_OXA−10_ variant carried on Int1 integrons. This indicates that these resistance genes can spread widely across different strains regardless of their genetic backgrounds, underscoring the significant role of these gene variants in the global dissemination of carbapenem resistance.

The present study offers compelling evidence of the evolutionary adaptation observed in carbapenem-resistant high-risk clones of *K. pneumoniae* in Egypt. In a previous multi-center pilot study on resistant *K. pneumoniae* conducted in Egypt, ST101 and ST147 emerged as the most predominant sequence types among the samples [54]. These were subsequently followed by ST383 and ST11, with each of the four sequence types being associated with specific sample types [54].

Notably, clones such as ST383 and ST147, were prevalent in this study and carried both *bla*_NDM−5_ and *bla*_OXA_ carbapenemases, in addition to a plethora of virulence genes. These high-risk clones were reported globally and in the Middle East [56, 57]. While ST383 had been previously reported in Egypt, exhibiting various variants of bla_CTX−M_, the present study reveals that ST383-K30 clones have acquired a wider range of carbapenemases and hypervirulence determinants. Similarly, ST147-K64, found in this study, has been globally identified as a hypervirulent clone associated with numerous invasive incidents and outbreaks in healthcare settings [57, 58]. Both scenarios present emerging potential threats, particularly in Egypt, a country already facing a significant burden of AMR [59].

In our current study, we identified nine different K-types. The diversity of capsule antigens among the isolates underscores their virulence variability and ability to evade host immunity. Consequently, the development of vaccines and diagnostic methodologies poses greater challenges [60]. Conversely, among the nine distinct O-antigen types of *K. pneumoniae*, O1-antigen was the most frequently observed.

Type 1 and type 2 fimbriae have distinct structures that help bacteria to adhere to biotic and abiotic surfaces and form biofilms. Type 1 fimbriae are encoded by *fimABCDEFGHIK* gene cluster, which were fully present among all the isolates and the MDR and hypervirulent reference strains. All the isolates, except one, carried the full gene cassette *mrkABCDFHIJ* encoding for type 3 fimbriae. This fimbrial type is associated with biofilm formation, which facilitates bacterial survival in harsh conditions, fosters AMR, and provides protection against host immune responses [61]. Siderophores, small compounds produced by microorganisms, bind iron with high affinity to ensure its availability for bacterial replication and colonization. *K. pneumoniae* produces various siderophores, including enterobactin, salmochelin, aerobactin, and yersiniabactin, each contributing to bacterial pathogenesis [62]. Gene clusters responsible for siderophore production were identified in all isolates, with enterobactin being the most frequently detected siderophore, followed by salmochelin and aerobactin. This suggests that the isolates employ diverse mechanisms to acquire iron for bacterial growth and survival.

The first hypervirulent metastatic *K. pneumoniae* strain was reported in China in 1986 [63]. However, the definition of hypervirulence remained controversial. Hypervirulence characterization of *K. pneumoniae* typically involves positive string test and specific capsule types (K1 and K2) associated with hypermucoviscosity. Yet, the string test lacks reliable positive predictive value for hypervirulence [64]. Additionally, K1 or K2 capsule types had low values of accuracy, sensitivity, and specificity as biomarkers for hypervirulence [65]. Furthermore, studies showed that hypermucoviscosity and hypervirulence are two independent phenotypes [66]. Thus, a widely accepted molecular and microbiological definition of hypervirulence remained undefined, despite its importance in reporting the hypervirulent strains causing infections [67]. Recently, a set of proposed hypervirulence genes designates a *K. pneumoniae* strain as hypervirulent when present. The gene set included hypermucoviscosity *rmpA* and/or *rmpA2*, aerobactin *iucA*, salmochelin *iroB*, and putative transporter *peg-344* [65]. However, in more recent research, aerobactin siderophore was proposed as a more reliable stable biomarker for hypervirulence due to its genomic stability [68].

Multiple factors determine the direction of the correlation between AMR and virulence in a certain bacterial strain. These include the host, the fitness of the bacterial species, the mechanisms of resistance and virulence, and the environmental niche accommodating the bacteria [69]. Previous studies have shown a negative correlation between AMR and hypervirulence in *K. pneumoniae* strains isolated from various specimens [70]. Nonetheless, multiple strains were reported to carry both AMR genes and hypervirulence determinants were reported [59, 71, 72]. The emergence of convergent multi-drug resistant hypervirulent *K. pneumoniae* strains represents a significant threat to the public health worldwide. Although classical MDR strains are known to be low-virulence organisms, recent studies reported an increase in hypervirulent drug resistant *K. pneumoniae* infections, especially in clinical settings of different countries [73]. Yet, few studies reported the full genomic characterization and spread of convergent strains in Egypt. In this study, seven putative convergent hypervirulent carbapenem-resistant isolates were reported based on their AMR and virulence profiles among the tested isolates. All these seven isolates were carbapenemase-producing, exhibited phenotypic resistance to both meropenem and imipenem, and carried one or more carbapenemase genes. These isolates carried the hypervirulence gene determinants, including *rmpA* and/or *rmpA2*,* iucABCD* gene cluster, and *peg-344.* Notably, these isolates were obtained from different specimens, including metastatic infections, a hallmark manifestation of hypervirulent *K. pneumoniae*, such as blood, cerebrospinal fluid, ascitic fluid and bed sores. This underscores the extensive pathogenic capability exhibited by these strains. Almost all these isolates were high-risk MDR clones, capable of widespread dissemination, such as ST11, ST14, ST383, and ST147. Additionally, to the best of our knowledge, we are reporting ST709 for the first time as a potentially high-risk clone carrying hypervirulence genetic determinants. At the K-type level, K2, K30, and K64 have been identified as hypervirulent K-types. Notably, we report K9 and K15 for the first time as potential hypervirulent capsule types alongside their MDR characteristics.

Due to its extremely plastic genome, *K. pneumoniae* tends to smoothly acquire plasmids and mobile genetic elements, thereby enhancing its adaptability for survival. For instance, IncL/M-type plasmids have been identified worldwide in Enterobacterales isolates from various origin [74]. They are epidemic resistance plasmids implicated in the large dissemination of specific genes encoding carbapenemase genes *bla*_NDM−1_ and *bla*_OXA−48_ [75]. In this study, most *bla*_OXA−48_ genes were encoded on IncL/M plasmids, as previously observed in Europe and the Mediterranean region [76]. Additionally, the IncFIB and IncHI1B plasmids, which are highly mobile, were found to carry several *bla*_NDM_ variants along with several other antibiotic resistance genes and an array of virulence genes, indicating their mobility and ability to integrate into different plasmids. The IncFIIK plasmids are linked to the worldwide spread of ESBLs and carbapenemases, especially in clinical isolates of *K. pneumoniae* [77].

Altogether, these plasmids constitute a rich niche of AMR determinants that may disseminate across diverse bacteria posing a substantial threat to human health. It is plausible that the seven studied isolates were initially MDR and subsequently acquired virulence plasmid that carried *iuc* gene cluster and *rmpA/rmpA2.* This hypothesis may be further supported by the fact that this gene set was found in the same plasmid contigs across the convergent isolates. There is a potential for the presence of mosaic plasmid carrying resistance and virulence genes such as IncFIB(Mar)/ IncFII(K) and IncFIB(Mar)/ IncHI1B hybrid plasmids [78].

The current study has some limitations. The sample size was small, and there was a lack of representative samples for each specimen type. Secondly, the short read length hindered the complete assembly of plasmids, making it challenging to study all the genes they harbored, as well as the flanking regions of insertion sequences and transposons. Furthermore, the characterization of hypervirulence was confirmed only at the genotypic level through in silico analysis.

## Conclusion

The study highlights the emergence of high-risk, extensively carbapenem-resistant *K. pneumoniae* strains with hypervirulence gene determinants in Egyptian clinical settings, aligning with the global spread of MDR-hypervirulent *K. pneumoniae* and posing a significant pandemic threat. Future directions include phenotypic and genotypic characterization of colistin resistance, validation of hypervirulence biomarkers. An in vivo infection model will be used to confirm the hypervirulence of the studied strains. Subsequently, long-read sequencing will be performed on confirmed convergent strains to identify hypervirulence plasmids and explore the genetic environment of resistance and virulence genes. Due to resource limitations, sequencing of additional isolates was not initially feasible, but expanding sequencing efforts remains a priority as resources become available.

## Electronic supplementary material

Below is the link to the electronic supplementary material.

Supplementary Material 1: Additional file 1.xsls holds supplementary tables along the manuscript, including sample accession numbers, MIC values, heavy metal resistance genes, virulence genes, plasmids, and insertion sequences detected within isolates genomes.

Supplementary Material 2: Supplementary figures S1, S2, and S3 illustrated the mobile genetic determinants within convergent strains.

Supplementary Material 3: Reviewer reports.

## Data Availability

Sequencing raw reads for the samples used in the study were deposited in the Sequence Read Archive database, where SRA accessions were provided in the supplementary table S1, additional file 1. The reads were a part of BioProject PRJNA906139. GenBank accession for the peg-344 putative transporter is MW911667.1. NCBI Genome accession for K. pneumoniae subsp. pneumoniae HS11286 is GCF_000240185.1. K. pneumoniae subsp. pneumoniae NTUH-K2044 genome accession is GCF_000009885.1.

## Abbreviations

AMR: Antimicrobial resistance
cgMLST: Core genome multi-locus sequence typing
CPS: Capsule polysaccharide
ECP: Escherichia coli common pilus
ESBL: Extended-spectrum β-lactamase
LMIC: Low- and middle-income countries
LPS: Lipopolysaccharide
MDR: Multi-drug resistant
MGE: Mobile genetic elements
MIC: Minimum inhibitory concentrations
MPF: Mate-pair formation proteins
PDR: Pan-drug resistant
ST: Sequence type
VFDB: Virulence factor database
XDR: Extensively-drug resistant
