# Supplementary figures and images for: Genomic dynamics of high-risk carbapenem-resistant Klebsiella pneumoniae clones carrying hypervirulence determinants in Egyptian clinical settings
Source: BMC Infect Dis. 2024 Oct 22;24:1193. doi: 10.1186/s12879-024-10056-1 (PMC11515790; doi:10.1186/s12879-024-10056-1)

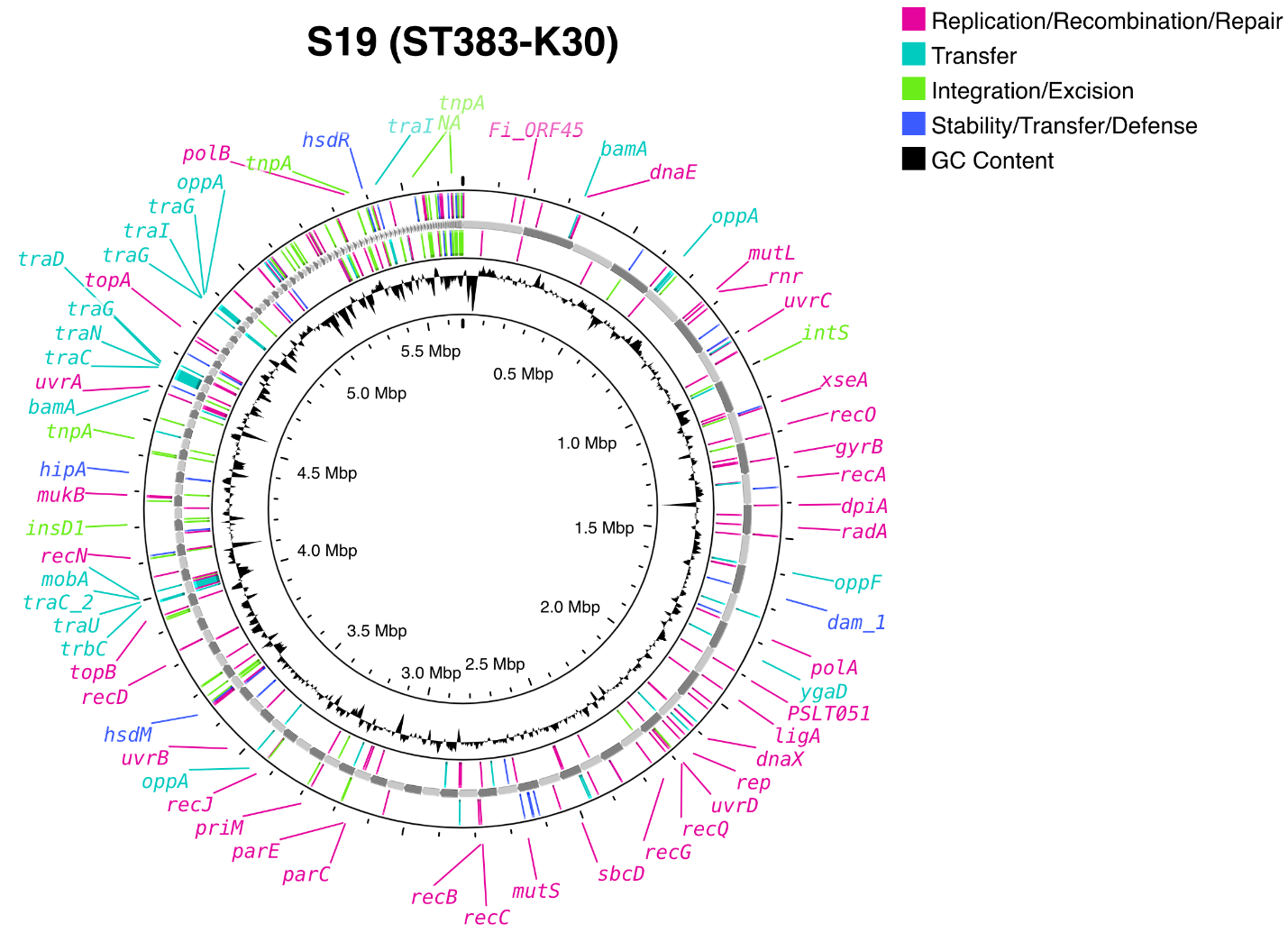


**Supplementary figure S1**


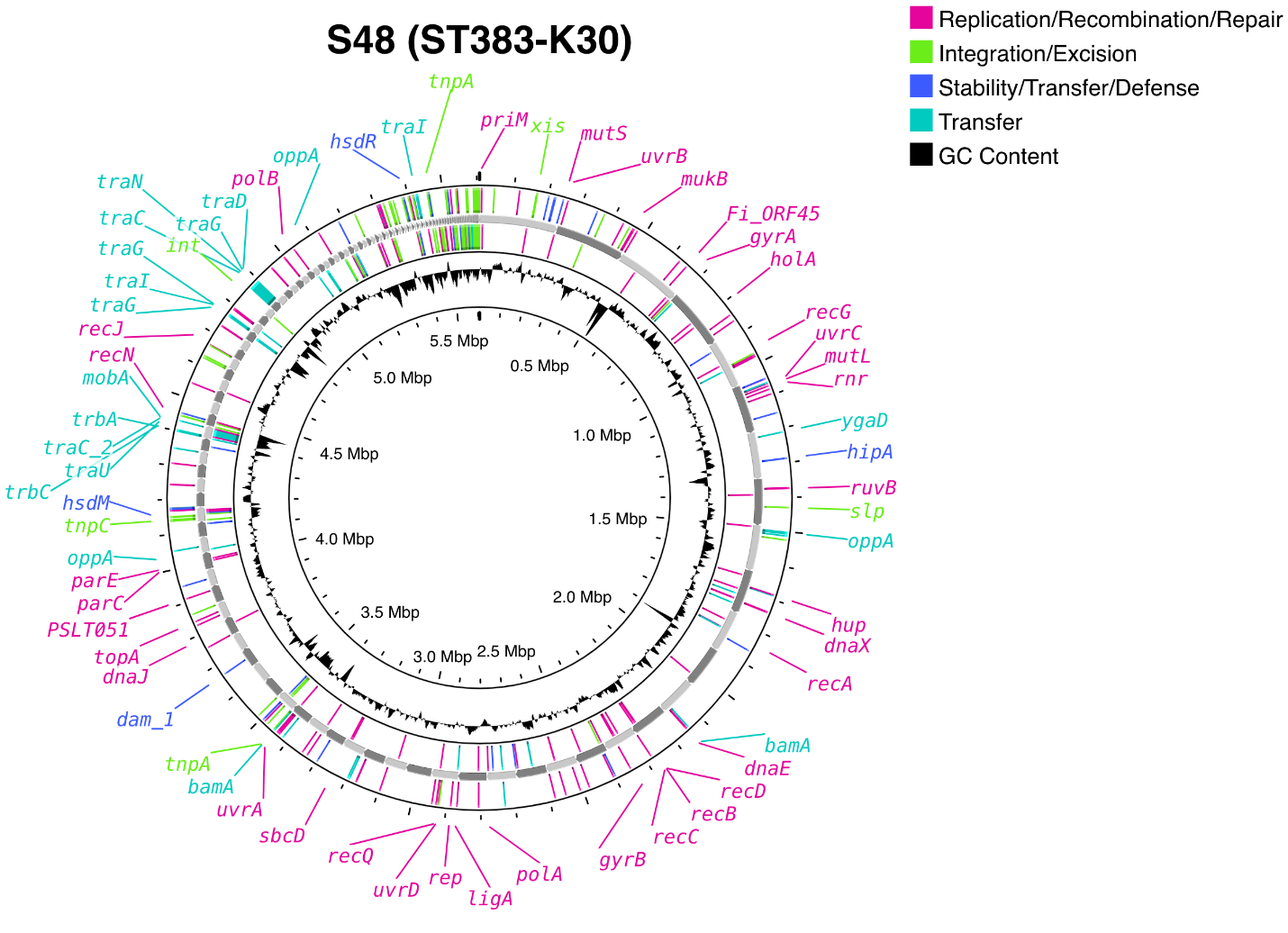


**Supplementary figure S2**


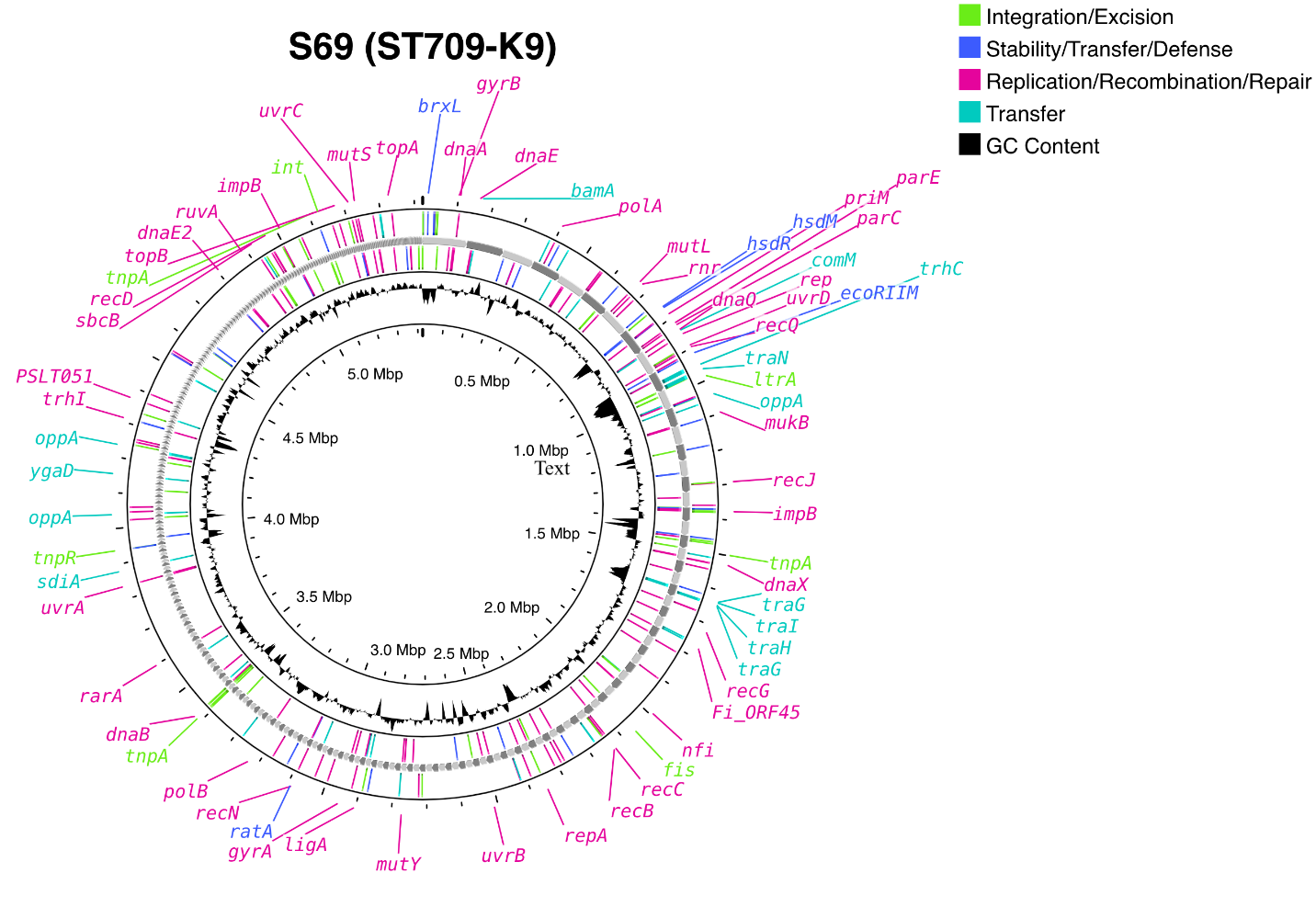


**Supplementary figure S3**

Supplement: Supplementary file 2 — Supplementary Material 2: Supplementary figures S1, S2, and S3 illustrated the mobile genetic determinants within convergent strains. [file 12879_2024_10056_MOESM2_ESM.docx]
