# Supplementary material for: Genomic dynamics of high-risk carbapenem-resistant Klebsiella pneumoniae clones carrying hypervirulence determinants in Egyptian clinical settings
Source: BMC Infect Dis. 2024 Oct 22;24:1193. doi: 10.1186/s12879-024-10056-1 (PMC11515790; doi:10.1186/s12879-024-10056-1)
Supplement: Supplementary file 3 — Supplementary Material 3: Reviewer reports. [file 12879_2024_10056_MOESM3_ESM.docx]

**Reviewer reports ‘Genomic Insights into the Emergence of Convergent Hypervirulent Carbapenem-Resistant Klebsiella pneumoniae in Egyptian Clinical Settings’**

**Version 1**

**Reviewer 1**

The manuscript by Abdelsalam et al is well written, very detailed, using appropriate methods, well structured and clearly stating the main findings. It is interesting that they characterize this collection, which although small, adds value to the knowledge of the epidemiology of CPE and represents a challenge when treating a possible infection. However, the manuscript is too long. It is not possible to publish a manuscript with so much detail, so unfortunately, despite how well written and detailed it is, it needs major revision. Also, I have several comments on other points.

- The introduction should be summarized in half. It is not necessary to detail the differences between classic and hypervirulent Kp (for example, lines 38-39, 45-50, 54-59, 63-67, 80-87... could be removed).

- The methods part is also very detailed, however they do not indicate what type of sequencing they have used (short-read or long-read or both??). It is important to make that clear and reference other works for other tools. It is not necessary to detail the functions of the bioinformatic tools used so much.

- They have not indicated whether the study was approved by an ethics committee.

-What have they used roary for? have done cgMLST, it's not necessary and I don't see the results.

- The full set of virulence genes to consider a Kp Hypervirulent includes iroB. The authors have analyzed it (the results are in supplementary material), but they must include it in the interpretation and in the figures. View manuscript (reference 82): Russo TA, Olson R, Fang C-T, Stoesser N, Miller M, MacDonald U, Hutson A, Barker JH, La Hoz RM, Johnson JR, for the Hypervirulent Klebsiella pneumoniae Investigator Group (HVKPIG). 2018. Identification of biomarkers for Differentiation of hypervirulent Klebsiella pneumoniae from classical K. pneumoniae. J Clin Microbiol 56:e00776-18. https://doi.org/10.1128/JCM.00776-18.

- The authors should review the phage annotation they have done with the mobileGO-db tool, in prokkse, since it is not a good db to annotate these elements. It must be confirmed with blast and other phage search tools. If they have it, put in methdos that was confirmed by Blast and by other tools (and specify them, briefly).

- The results section should also be summarized. The tables and figures contain a lot of information, so it is not necessary to go into so much detail in the manuscript. Each section of results should be summarized in half and only the most relevant results should be counted.

- The OXA-1 and OXA-9 genes are not carbapenemases. This ruling is very important. OXA-1 is an ESBLs and OXA-9 is a beta-lactamase. The authors should review the entire text, recalculate the percentages of CPEs detected and modify the figures. If you have questions about beta-lactamase genes, you can consult the page: http://bldb.eu/

- Have the authors analyzed the unknown mutations in OmpK35 and OmpK36? Pointfinder allows you to do the analysis looking for only known mutations or all. The authors should review everything, since some strains could have mutations that give rise to stop codons or changes in the reading frame, which would affect the functionality of the porin.

- The analysis of resistance genes that affect other groups of antimicrobials should be summarized in a single section. There is no need to give so much detail (the gene content is shown in the figures), you just have to highlight the most relevant.

- Also summarize the analysis of virulence factors and MGEs.

-In the discussion, it is not necessary to expand so much on previous studies with Kp-ESBLs, since they are not the objective of this study (lines 450-468).

- Line 616: new antihypervirulence antimicrobial therapies??

**Reviewer 2**

The authors presented a genomic study on the characterization of 19 carbapenem-resistant Klebsiella pneumoniae from Egypt. Although the manuscript is well written, the results section can be focused without redundant information from the Figures. One of the main weakpoints is the lack of denominator and the selection strategy, which is important for interpreting the results in a local epidemiological context.

Specific comments:

Line 47: Please cite original articles to support the claim that hvKP contributes to increased mortality. Citing a review article is not sufficient.

Line 89: The term "high-throughput" sequencing is not accurate and seems out of place given that you only characterised 19 isolates in this study.

Introduction: although there are some data for more susceptible hvKp, there are no data to suggest that the clinical course of infections with MDR hvKp is more severe than with MDR-Kp, hvKp or classic Kp. You may wish to comment on this. Therefore, I would disagree with lines 91-93 that the genomic characterisation of hvKP contributes to the management of severe infections... If you claim this, then please explain the difference in the clinical management of Kp, hvKp or MDR hvKp. The clinical management of MDR versus non-MDR classic KP may be different, but there are no data to support your claims for hvKp.

Line 96-97: 19 hvKp out of how many in total? Without a denominator the data is not very useful What about duplicate isolates from one patient and multiple localisations, were these excluded?

Line 118: What is the definition of low quality raw reads? It should be provided without the need to look at other publications. This is important data for WGS studies.

Line 120: SPAdes version should be provided. What about recombination sites, are they included in the bioinformatic analysis?

Methods: please provide any pipelines or software used for the analysis (e.g. Prokka, Resfinder, etc).

Results: What was the search and inclusion strategy for the 19 isolates? Please give the number of carbapenemase producing Kp (non-duplicate) and mention how the 19 were selected? How many did you miss or were you able to include all CP-Kp from the mentioned study period?

Results (lines 196-200): It's enough to mention that all were beta-lactam resistant. I don't think it's necessary to include all MICs.

Results (ARGS), lines 235 to 367 - please do not repeat all the information from Figure 1 in text form - this is not necessary unless it is important, but there is no need to list the prevalence of each gene separately. This information is summarised in Figure 1.

Discussion is good but too long, please keep the discussion to the main points.

Limitations: The "convergent" Kp were characterised by genotype only. Usually some phenotypic confirmation, such as siderophore production, in vivo infection model (Galleria mellonella or mouse model) is required to confirm the hvKp.

Line 615-616: I disagree, the study showed no correlation between clinical outcomes and hypervirulence/convergence. It is speculative that antivirulence drugs are needed.

Conclusion: Why should colistin resistance be a direction for future study and not included here? The isolates are available, the genomes are sequenced and the AST can easily be done with the number of isolates included in the study (not that many). This needs to be justified, why this was not included in this manuscript. Were colistin resistance genes (mcr?) detected? I recommend adding colistin AST to this manuscript.

Line 492 - please specify which OXA is meant. Not all oxacillinases can hydrolize carbapenems. Also line 499

>6000 words on a genomic study with only 19 isolates is a bit much, please consider reducing redundant passages and focus on the main points.

**Version 2**

**Reviewer 2**

„Unfortunately, there are no clear statistics on the number of K. pneumoniae infections in Egypt, either in clinical or community settings. However, in our previous study on a pool of Gram-negative bacteria isolated from hospitals in Egypt, 46 samples were confirmed to be K. pneumoniae. Out of these, 28 samples were randomly selected for short-read sequencing. Based on carbapenemase production and thresholds set for sequence completeness and contamination (Lines 176-177), the genomes of the 19 mentioned isolates were further detailed in the manuscript. To avoid confusing the reader, we mentioned that our inclusion criteria were carbapenemase production and phenotypic resistance to meropenem and imipenem (Lines 72-74).“ The answer is not adequate. Please include background information on how the strains were collected (strategy) so that the information is plausible to the reader. 46 K pneumoniae from clinical isolates or colonisation? From which year? What was the search strategy for the previous study? What was the reason for not sequencing the remaining? Line 70: Late 2020 and early 2021? Please specify months! If you randomly select 28 for sequencing, why are you only including 19 in this study? I find it very difficult to follow the sampling strategy. A molecular epidemiology study needs a clean design and the inclusion strategy should be plausible.
